# A Study on the Dynamic Switching Characteristics of p-GaN HEMT Power Devices

**DOI:** 10.3390/mi15080993

**Published:** 2024-07-31

**Authors:** Chen Fan, Haitao Zhang, Huipeng Liu, Xiaofei Pan, Su Yan, Hongliang Chen, Wei Guo, Lin Cai, Shuhua Wei

**Affiliations:** 1 School of Information Science and Technology, North China University of Technology, Beijing 100144, China; fanchen@mail.ncut.edu.cn; 2 Beijing Huafeng Test & Control Technology Co., Ltd., Beijing 100094, China; huipeng.liu@accotest.com (H.L.); su.yan@accotest.com (S.Y.); hongliang.chen@accotest.com (H.C.); linda.cai@accotest.com (L.C.); 3 Huafeng Test & Control Technology (Tianjin) Co., Ltd., Tianjin 300457, China; 4 Ningbo Institute of Materials Technology and Engineering, Chinese Academy of Sciences, Ningbo 315201, China; guowei@nimte.ac.cn; 5 Ningbo Daxin Semiconductor Co., Ltd., Ningbo 315400, China; 6 State Key Laboratory of Power Transmission Equipment Technology (Chongqing University), Shapingba District, Chongqing 400044, China; xiaofei_pan@anst.crmicro.com

**Keywords:** p-GaN HEMT, dynamic switching, capacitance, trap

## Abstract

This study employs an innovative dynamic switching test system to investigate the dynamic switching characteristics of three p-GaN HEMT devices. The dynamic switching characteristics are different from the previous research on the dynamic resistance characteristics of GaN devices, and the stability of GaN devices can be analyzed from the perspective of switching characteristics. Based on the theory of dynamic changes in threshold opening voltage and capacitance caused by electrical stress, the mechanism of dynamic switching characteristics of GaN HEMT devices is studied and analyzed in detail. The test results have shown that electrical stress induces trap ionization within the device, resulting in fluctuations in electric potential and ultimately leading to alterations in two critical factors of the dynamic switching characteristics of GaN HEMT devices, the parasitic capacitance and the threshold voltage. The dynamic changes in capacitance before and after electrical stress vary among devices, resulting in different dynamic switching characteristics. The test system is capable of extracting the switching waveform for visual comparison and quantitatively calculating the changes in switching parameters before and after electrical stressing. This test provides a prediction for the drift of switch parameters, offering pre-guidance for the robustness of the optimized application scheme.

## 1. Introduction

As a representative material of third-generation semiconductor materials, gallium nitride (GaN) exhibits superior material qualities, including larger bandgap width, higher electron mobility, and higher electron saturation speed, outperforming its Silicon counterparts. Recently, GaN HEMT devices have demonstrated great potential in power electronics applications [1]. However, the performance degradation of GaN HEMT devices under electrical stress confines their further application and commercialization [2]. Previous research have predominantly focused on dynamic resistance characteristics, threshold opening voltage ($V_{TH}$) drift, and Capacitance-Voltage threshold drift characteristics [3], while the change in the switching characteristics of the device before and after electrical stress has not been comprehensively analyzed.

While the high switching speed of GaN HEMT devices enables the power electronic systems to achieve higher energy conversion efficiency and energy density [4], it also poses certain challenges to the reliability of systematic applications. In the power electronics application of GaN HEMT devices, the switching frequency continues to increase, and the dead time constantly decreases. Significant variations in the switching parameters of the device after electrical stress could easily lead to instability in system operation [5]. Previous studies primarily utilize the C-V threshold drift test method to characterize the AC parameters of the device [6,7]. However, the large test delay and the long test cycle that takes several seconds or even tens of seconds make the C-V threshold drift test method unsuitable for achieving high-frequency, high-speed, and high-precision mass production testing. Previously, we have proposed a dynamic capacitance measurement method for GaN-based devices that detects the capacitance changes at fixed bias voltage before and after electrical stress, realizing a fast continuous characterization of the capacitance parameters with a delay as low as 100 ms at a single bias voltage after electrical stress [8]. Nonetheless, the change in capacitance parameters at a single bias voltage cannot fully characterize the change in the AC characteristics of the device.

In this study, we propose an unprecedented complete test method for “dynamic switching characteristics” and achieve stable and repeatable results. Unlike dynamic resistance testing, which focuses on the on-resistance, dynamic switch testing compares the switching parameters including current rise time and turn-on delay time, etc., before and after electrical stress. Currently, no general-purpose equipment is capable of performing this test. Based on the STS8200 test platform of Accotest Company (Beijing, China), we initiate a specialized test system, realizing zero-delay post-stressing switching and the precise extraction of switching parameters before and after electrical stress. By comparing the switching parameters before and after electrical stress, the changes in switching characteristics can be accurately characterized.

Based on this testing system, three p-GaN HEMTs were tested dynamically. The switching parameters changed after electrical stress, and different devices showed different changing trends. Combined with our previous research on dynamic capacitance [8] and the parasitic parameter model of p-GaN HEMT devices, we have performed a comprehensive mechanism analysis of the corresponding dynamic variation characteristics of the observed switching parameters. The traps in the device exhibit different ionization states after being excited by electrical stress, and the traps cannot be restored to electrical neutrality within a short time after being stressed, resulting in significant changes in the parasitic capacitance parameters and $V_{TH}$ of the devices [8]. The switching characteristics of power devices are closely related to parasitic capacitance and $V_{TH}$ [9,10], and the dynamic changes in capacitance and $V_{TH}$ ultimately lead to dynamic changes in the switching parameters of the device. In this paper, we propose and implement a novel dynamic switching test method for p-GaN HEMT power supplies, enabling the accurate measurement of the changes in switching parameters before and after device stress. Based on the mechanism analysis of the test results, it provides a new approach for the performance evaluation and screening of p-GaN HEMT devices.

## 2. Dynamic Switch Test of GaN Device

The dynamic resistance test of GaN HEMT devices involves measuring and comparing the conduction internal resistance ($R_{ON}$) parameters before and after the electrical stress. In contrast, the dynamic switching characteristic records and compares the switching parameters of the device before and after electrical stress. 

**Figure 1:**
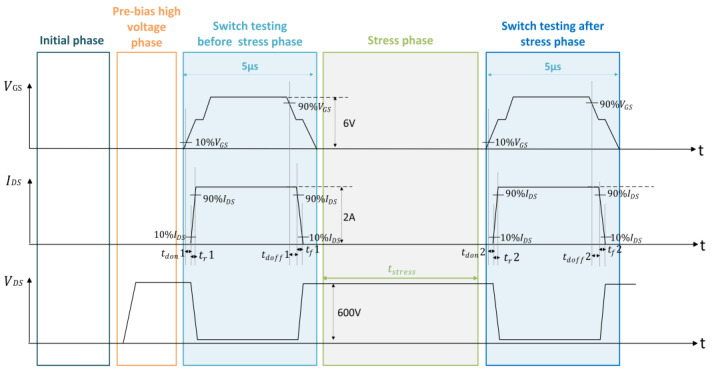
Dynamic switch test sequence diagram with STS8200.

In this study, the resistive load test method was used to eliminate the phase difference between bus voltage and bus current, which can study the switching characteristics of the device more conveniently and accurately [11]. As shown in Figure 1, we identified key switching time parameters, including turn-on delay time ($t_{don}$), current rise time ($t_{r}$), turn-off delay time ($t_{doff}$), and current fall time ($t_{f}$). The sequence diagram used in this study is shown in Figure 1. At the initial off state, no electrical stress is applied across the device, and no current flows. Before the first switching test, to simulate the switching process of the device at a high busbar voltage, a pre-bias high voltage consistent with the electrical stress voltage is applied to the device for less than 1 ms. The pre-bias high voltage applies electrical stress, but it has been verified that this 1 ms electrical stress does not alter the switching parameters of the device. After the pre-bias high voltage stabilizes, the device undergoes a 5 μs switching parameter test, and then, the device is subjected to continuous high-voltage electrical stress for a specified duration. Following the end of the electrical stress, a zero-delay switching test is applied to the device. The comparison of switching parameters before and after the electrical wraps up the dynamic switching test.

**Figure 2:**
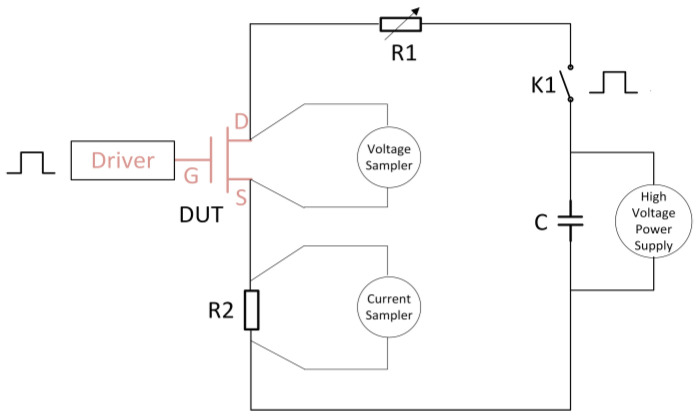
Dynamic switch test circuit diagram.

GaN devices exhibit short turn-on and turn-off times. To accurately capture the switching characteristics of GaN devices, it is essential to reduce loop parasitic distribution parameters, improve test communication speed, and utilize high-resolution and high-performance oscilloscopes. Based on the AccoTest STS8200 test platform, we optimized the test circuit design and significantly reduced the loop parasitic distribution parameters. The gate drive circuit of the device-under-test (DUT) includes a high-voltage isolated driver chip [12] to achieve a controllable turn-on and turn-off of the DUT. As shown in Figure 2, the test circuit adopts a resistive load design, through which the desired current can be achieved easily by adjusting R1. By controlling switch K1, the high voltage in the circuit can be interrupted and reconnected, and the electrical stress can be terminated before and after the test. A high-speed high-precision Tektronix MSO58 high-speed multi-channel oscilloscope was employed to measure the GS terminal voltage and GD terminal voltage in real time. The real-time loop current was monitored by detecting the voltage on the current-monitor resistor R2. The test system is capable of rapidly switching 600 V electrical stress, monitoring the entire process of voltage stress lasting over 10 s and capturing the switching parameters with high precision during the high-speed switching process.

Three 650 V voltage standard devices labeled A/B/C from different manufacturers with various materials and fabrication procedures were selected and tested. These three devices are common p-GaN HEMT technology and do not use a special structural design. The typical values of on-resistance at room temperature are 240 mΩ, 130 mΩ, and 40 mΩ, respectively. For all DUTs, the gate drive was driven to a maximum voltage of 6 V by a constant 10 mA current, the $V_{DS}$ voltage of the switch test was 600 V, and the current of the device during the switch test was 2 A.

## 3. Experimental Results and Analysis

### 3.1. Experimental Results

**Figure 3:**
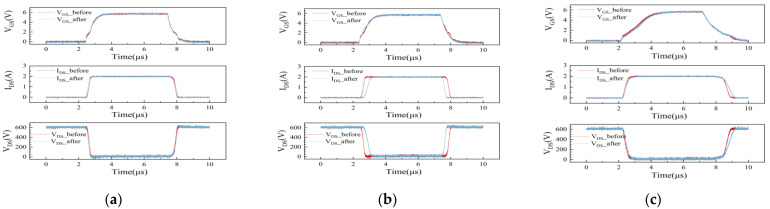
Comparison of switch waveforms before and after electrical stress of sample A/B/C. (**a**): Sample A; (**b**): Sample B; (**c**): Sample C.

The A/B/C devices were subjected to 600 V high-voltage continuous electrical stress for 10 s, and the DUTs were tested according to the timing protocols. The PicoScope 6824E oscilloscope (Cambridgeshire, UK) was employed to capture the test results of the $V_{GS}$/$V_{DS}$/$I_{DS}$ before and after the electrical stress. Table 1 presents a comparison of the typical values for dynamic switching parameters among the three devices. The waveforms before and after electrical stress for each parameter of each device are displayed in a single window for comparative analysis, as depicted in Figure 3, combined with waveform and data. The parameters of sample A exhibit minimal change after electrical stress, with only a slight reduction in the turn-on delay time $t_{don}$. In contrast, the parameters of sample B show significant changes after electrical stress. During the turn-on phase, the turn-on delay time $t_{don}$ increases after electrical stress, and the current rise time $t_{r}$ increases. In the turn-off phase of sample B, the turn-off delay time $t_{doff}$ decreases after electrical stress, while the current fall time $t_{f}$ remains relatively stable. In the turn-on phase of sample C, the parameters barely changes significantly. The turn-off delay time $t_{doff}$ exhibits no significant changes, but the current fall time $t_{f}$ increases notably. The test waveforms from the three samples align with the test results.

**Figure 4:**
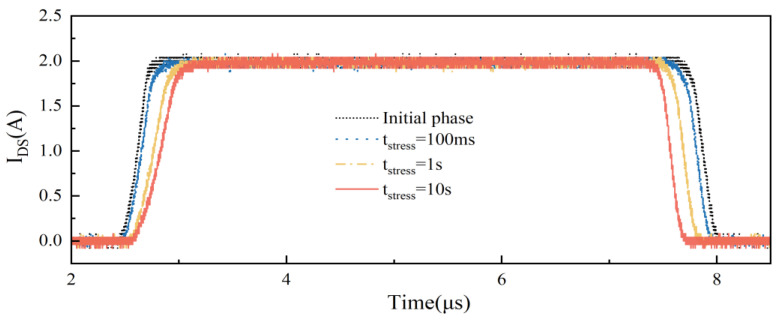
Dynamic switching characteristic curve of sample B.

To investigate the trend in dynamic switching characteristics of the DUTs, a 100 ms~10 s gradient electrical stress was applied. Figure 4 shows the switching waveform of sample B’s $I_{DS}$ in its initial state, after 100 ms of electrical stress, after 1 s of electrical stress, and after 10 s of electrical stress. To ensure complete device recovery between each test, the interval between each test was maintained for a minimum of 24 h. As illustrated in Figure 4, the change in switching characteristics of the device shows a monotonic trend with increasing electrical stress.

To further analyze the characteristics of the device, the parameters were extracted and calculated by the STS8200 test system. Five consecutive tests were performed on the same sample at intervals exceeding 24 h to validate the accuracy of the test system data. Data differences of ±5% or less were observed, representing the high accuracy, high reliability, and stable testing of the STS8200 test system. With the help of the fast computing capability of the STS8200 test system, $t_{don}$ and $t_{r}$ related to the opening process and $t_{doff}$ and $t_{f}$ related to the turn-off process before and after the electrical stress were quantitatively extracted according to the parameter protocols shown in Figure 1. Then, the dynamic change in switching characteristics of the device was quantified by calculating the rate of change before and after electrical stress.

**Figure 5:**
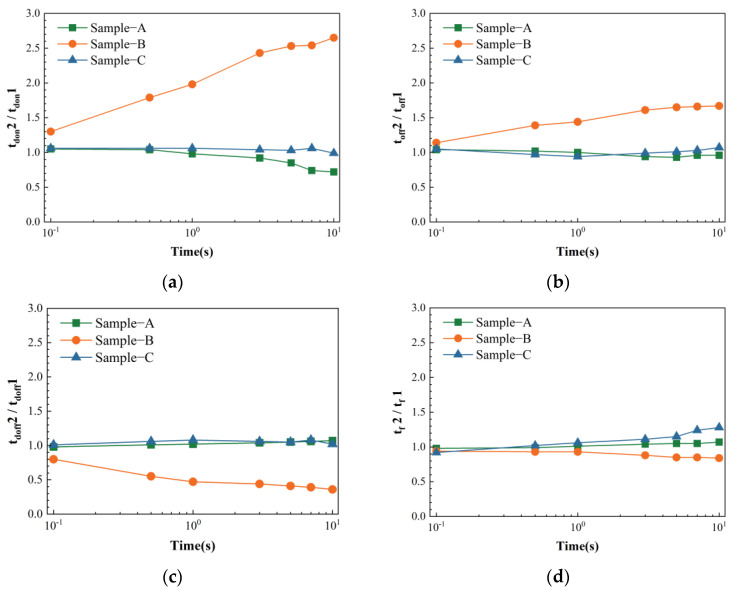
Quantitative comparison of switching parameters of A/B/C sample before and after electrical stress with stress time. (**a**): turn-on delay time; (**b**): rise time; (**c**): turn-off delay time; (**d**): fall time.

Figure 5 presents the trend in all parameters of the three devices with electrical stress time. In sample A, with increasing electrical stress time, $t_{don}$ decreases, while other parameters remain stable. In sample B, with the increase in electrical stress time, the $t_{don}$ and $t_{r}$ of sample B both exhibit an upward trend, while the $t_{doff}$ decreases significantly, and $t_{f}$ presents negligible changes. For sample C, as the electrical stress time increases, the relevant parameters $t_{don}$, $t_{r}$, and $t_{doff}$ show minimal variations, but $t_{f}$ increases significantly. The summary above indicates that as the electrical stress time increases, the rate of parameter change in the DUT increases, the device parameters exhibit a consistent monotonic trend, and the quantitative trend aligns with the waveform comparison directly collected by the oscilloscope.

### 3.2. Mechanism Analysis

The test results of dynamic switches for the three devices are used as the basis for conducting mechanism analysis. The parasitic capacitance parameters of GaN HEMT devices are closely related to the switching characteristics of the devices. The dynamic changes in the switching parameters before and after electrical stress indicate that the parasitic capacitance parameters of the devices change significantly. Previously, we have conducted studies on the dynamic characteristics of the parasitic capacitance of GaN devices [8]. Results indicate that the trap ionization of GaN devices after stressing cannot restore electrical neutrality immediately after the electrical stress is removed, resulting in potential differences within the device and ultimately leading to various changes in the parasitic capacitance of the device.

**Figure 6:**
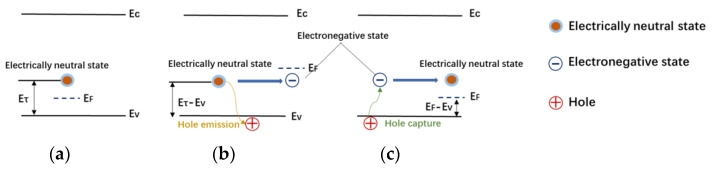
Schematic of acceptor-trap-induced capture and emission of free carrier. (**a**): Initial phase; (**b**): Stress phase; (**c**): Recovery phase.

Through the capture and emission of free carriers, the charge-trapping effects can affect the electrical potential inside the device, as exemplified in Figure 6 for acceptor traps. As shown in Figure 6b, the deep-level acceptor traps in the device release holes under a high electric field, yielding negative space charges in the buffer layer [13,14]. These negative space charges result in a reduction in the potential. When entering the recovery phase, these negatively charged acceptor traps accumulating in the GaN device start to capture holes from the valence band, recovering into an electrically neutral state, as depicted in Figure 6c.

**Figure 7:**
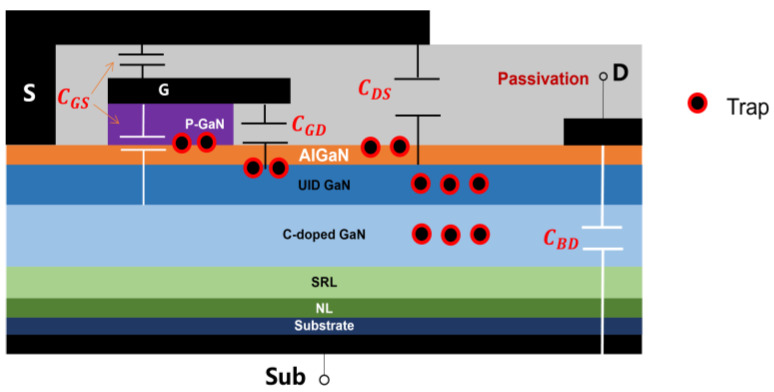
Schematic diagram of the parasitic capacitance distribution of a p-GaN HEMT device.

A typical p-GaN HEMT device structure diagram is demonstrated in Figure 7, with the identification of the main parasitic capacitance distribution inside the device and the location of possible traps. During the growth stage of materials, numerous traps are introduced into the UID layer and the carbon-doped layer, while interface traps can be presented at the AlGaN/GaN interface and the AlGaN/passivation layer interface, and the p-GaN gate introduces a further amount of traps [15,16].

The switching parameters of GaN HEMTs are closely related to their parasitic capacitance parameters. The main capacitance parameters include input capacitance $C_{iss}$, output capacitance $C_{oss}$, and Miller capacitance $C_{rss}$. $C_{iss}$ consists of $C_{GS}$ and $C_{GD}$; $C_{oss}$ consists of $C_{GD}$ and $C_{DS}$; $C_{rss}$ is from $C_{GD}$, also known as the Miller capacitor. The stability of Miller capacitors directly determines the device performance and stability, significantly affects the stability of the switching parameters of the device [17,18,19]. The effect of electrical stress on the parasitic capacitance was further analyzed with the results from the dynamic switching test.

**Figure 8:**
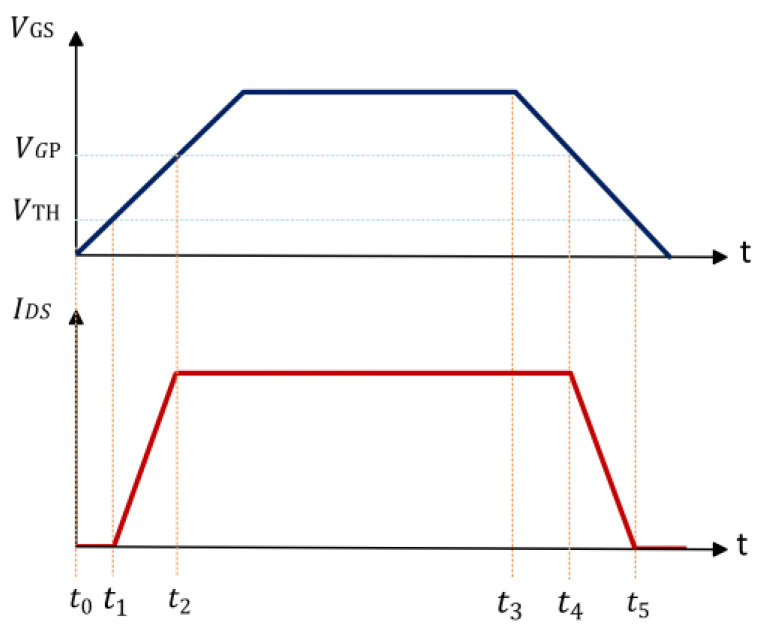
Schematic diagram of the switch curve.

Figure 8 illustrates the timing diagram of $V_{GS}$ and $I_{DS}$ in the switching process of a p-GaN HEMT device. The main parameters of the switching process of the device include turn-on delay time $t_{don}$, current rise time $t_{r}$, turn-off delay time $t_{doff}$, and current fall time $t_{f}$. Since the voltage and current are completely time-reversed for the resistive load test, there exists no phase difference between bus voltage and bus current in the inductive load test. The formula for calculating each time parameter is as follows [20]:

(1) $$t_{don}=t_{1}-t_{0}\sim \frac{C_{GS}+C_{GD}}{J_{G}}V_{TH}$$

(2) $$t_{r}=t_{2}-t_{1}\sim \frac{C_{GS}+C_{GD}}{J_{G}}\left(V_{GP}-V_{TH}\right)$$

(3) $$t_{doff}=t_{4}-t_{3}\sim \frac{C_{GS}+C_{GD}}{J_{G}}\left(V_{GS}-V_{GP}\right)$$

(4) $$t_{f}=t_{5}-t_{4}\sim \frac{C_{GD}}{J_{G}}\left(V_{GP}-V_{TH}\right)$$

where $\;C_{GS}$ is the gate-source capacitances; $C_{GD}$ is the gate-drain capacitances; $J_{G}$ is the gate current density; $V_{TH}$ is the threshold voltage; $V_{GP}$ is the gate voltage when the Miller capacitor is full during the device turn-on process; and $V_{GS}$ is the bias voltage set by the gate driver to the gate when the device is turned on.

Voltage stress significantly impacts trap ionization in gallium nitride devices, which leads to changes in the internal potential of gallium nitride devices. After removing the electrical stress, the trap remains electrically ionized for a prolonged time duration, resulting in changes in the $V_{TH}$ and parasitic capacitance of the devices [21,22,23,24]. Various types of traps are present inside GaN HEMT devices, each demonstrating different potential states after ionization. The acceptor trap ionizes negatively, while the donor trap ionizes positively. Due to different fabrication conditions, traps vary in locations and types for different devices, which affects the dynamic switching characteristics of the device differently after being excited by electrical stress. Therefore, the three devices from different manufacturers show unique dynamic switching characteristics.

The parameters of sample A shown in Figure 3 exhibit negligible changes before and after the electrical stress, except for a slight reduction in the turn-on delay time $t_{don}$. According to Formulas (1) and (2), the device may show a slight drift in $V_{TH}$. In the on-stage of sample B, the turn-on delay time $t_{don}$ and the current rise time $t_{r}$ increase. The turn-off delay time $t_{doff}$ decreases during the off stage after stressing, while the current fall time $t_{f}$ remains relatively stable. According to Formulas (1)–(4), the $V_{TH}$ and $V_{GP}$ of the device may increase after the electrical stress. The parameters of sample C in the opening stage and the turn-off delay time $t_{doff}$ in the turning-off stage show negligible changes after stressing, but the current fall time $t_{f}$ at the turn-off stage increases significantly. It is likely that $C_{GD}$ of the device may increase significantly after the electrical stress. The variation in the switching parameters of GaN devices following voltage stress indicates that the threshold voltage and parasitic capacitance of GaN devices are influenced by the applied voltage.

## 4. Conclusions

In this study, a dynamic switch test system based on the AccoTEST STS8200 test system has been successfully developed, accurately assessing the variations in switching parameters and the waveforms of the switching process exhibited by the device under test. Three devices from different manufacturers have been tested and analyzed, and the results illustrate a distinct dynamic change trend. Through the dynamic switching test of different devices, the reason for the switching characteristic drift of GaN devices after electrical stress is discussed. The experimental results demonstrate that the dynamic changes in the switching characteristics of GaN devices primarily arise from the excitation of trapped ionization by applied electric stress. This excitation leads to the capture or release of charge carriers, particularly because the ionization state of traps cannot be immediately restored after removing the electric stress, thus continuing to impact internal electric field distribution and causing potential fluctuations. This effect directly leads to significant changes in the key parameters of the device—threshold voltage ($V_{TH}$) and parasitic capacitance, which affect the overall performance of the device. However, the type and distribution of traps within GaN devices vary among different manufacturers, leading to distinct variations in $V_{TH}$ and parasitic capacitance, thereby resulting in diverse dynamic switching characteristics following electrical stress. The dynamic switching test method for p-GaN HEMT devices designed and implemented in this paper can accurately test various switching parameters, preliminarily revealing the deep underlying mechanism of the instability of p-GaN HEMT device performance under voltage stress. This discovery not only provides a valuable theoretical basis for optimizing GaN device design and enhancing device reliability, but also provides an important reference for the performance evaluation and selection of GaN HEMT devices in high-frequency electronic applications.

## Figures and Tables

**Table 1:** Comparison of switching time before and after electrical stress of A/B/C devices.

Sample		$t_{don}$/ns	$t_{r}$/ns	$t_{doff}$/ns	$t_{f}$/ns
A	Before	104.128	161.931	336.265	183.085
	After	75.448	155.26	359.707	194.573
B	Before	129.979	157.143	402.487	157.85
	After	344.494	262.642	146.886	132.224
C	Before	111.293	282.278	1287.868	415.662
	After	110.429	301.407	1319.466	530.22

## Data Availability

Data are contained within the article.
